# Detection of SARS-CoV-2 Δ426 ORF8 Deletion Mutant Cluster in NGS Screening

**DOI:** 10.3390/microorganisms11102378

**Published:** 2023-09-23

**Authors:** Riccardo Cecchetto, Emil Tonon, Nicoletta Medaina, Giona Turri, Erica Diani, Pier Paolo Piccaluga, Angela Salomoni, Michela Conti, Evelina Tacconelli, Anna Lagni, Virginia Lotti, Mosé Favarato, Davide Gibellini

**Affiliations:** 1 Microbiology Section, Department of Diagnostic and Public Health, University of Verona, 37134 Verona, Italy; riccardo.cecchetto@univr.it (R.C.); emil.tonon@univr.it (E.T.); anna.lagni@univr.it (A.L.); virginia.lotti@univr.it (V.L.); davide.gibellini@univr.it (D.G.); 2 UOC Microbiology Unit, AOUI Verona, 37134 Verona, Italy; nicoletta.medaina@aovr.veneto.it (N.M.); giona.turri@aovr.veneto.it (G.T.); 3 Hematopathology Section, Department of Experimental, Diagnostic, and Experimental Medicine, Bologna University, 40126 Bologna, Italy; pierpaolo.piccaluga@unibo.it; 4 Istituto Zooprofilattico Sperimentale delle Venezie, Legnaro, 35020 Padua, Italy; asalomoni@izsvenezie.it; 5 Infectious Diseases Section, Department of Diagnostic and Public Health, University of Verona, 37134 Verona, Italy; michela.conti@univr.it (M.C.); evelina.tacconelli@univr.it (E.T.); 6 Molecular Diagnostics and Genetics, AULSS 3 Serenissima, 30174 Venice, Italy; mose.favarato@aulss3.veneto.it

**Keywords:** SARS-CoV-2, genomic surveillance, NGS, deletion, variants

## Abstract

Next-generation sequencing (NGS) from SARS-CoV-2-positive swabs collected during the last months of 2022 revealed a large deletion spanning ORF7b and ORF8 (426 nt) in six patients infected with the BA.5.1 Omicron variant. This extensive genome loss removed a large part of these two genes, maintaining in frame the first 22 aminoacids of ORF7b and the last three aminoacids of ORF8. Interestingly, the deleted region was flanked by two small repeats, which were likely involved in the formation of a hairpin structure. Similar rearrangements, comparable in size and location to the deletion, were also identified in 15 sequences in the NCBI database. In this group, seven out of 15 cases from the USA and Switzerland presented both the BA.5.1 variant and the same 426 nucleotides deletion. It is noteworthy that three out of six cases were detected in patients with immunodeficiency, and it is conceivable that this clinical condition could promote the replication and selection of these mutations.

## 1. Introduction

SARS-CoV-2 is the etiologic agent of the current COVID-19 pandemic, and it has been classified in the Coronaviridae family [1]. The genome of SARS-CoV-2 is represented by a 30 kb single-stranded, positive-sense RNA virus characterized by six functional open-reading frames (ORFs) represented by replicase (ORF1a/ORF1b), spike (S), envelope (E), membrane (M), and nucleocapsid (N). In addition, seven ORFs encoding accessory proteins are detectable between the structural genes [2]. Although the presence of the viral endonuclease nsp14 decreases the mutation rate induced by the lack of proofreading activity of viral RNA-dependent RNA polymerase [3], SARS-CoV-2 displays a consistent mutation rate in its genome [4]. Moreover, genome recombination is a classic feature of Coronaviridae and therefore of SARS-CoV-2 [5]. The ability of SARS-CoV-2 to either mutate or recombine its genome elicits a clear advantage in viral biology by overcoming the selective pressure of the environment and, more precisely, of the immune system. Sequencing analysis of the SARS-CoV-2 genome isolated during the pandemic demonstrated the continuous onset of variants to support consistent viral spreading. Interestingly, the current circulating variants have exhibited a lower clinical impact than the original SARS-CoV-2 Wuhan strain [6] as well as an increase in escape from the immune response, as demonstrated by both the failure of several monoclonal antibody treatments [7] and decreased vaccine effectiveness [8]. 

The variant classification is mainly related to S gene mutations [9] involved in structural modifications in protein S. This protein recognizes the angiotensin-converting enzyme 2 (ACE2) cell receptor, which determines, together with co-receptors such as transmembrane serine protease 2 (TMPRSS2), viral entry into the cell [10]. Subsequent studies [11,12] showed that even specific mutations in other viral genes can still determine changes in viral replication performance, but the real impact remains to be elucidated. Furthermore, viral strains might recombine, thus determining the onset of new viral variants. These variants include, for instance, the XE, XF, and more recently, XBB strains [13], thus indicating that cellular coinfection with different viral lineages can determine the emergence of new viruses with mixed characteristics that might exert a significant impact on the pathogenesis and transmission of SARS-CoV-2 [14]. Intriguingly, the onset of new variants originating from mutations and/or recombination events is mainly associated with persistent infections, for example, in immunocompromised patients [15,16]. In these cases, the persistence of infection elicits many rounds of viral replication with an increased probability of genome mutation and/or recombination and the onset of a novel variant [17,18,19]. Interestingly, the appearance of Omicron lineage is likely related to persistent infection in an immunocompromised patient, generating a large number of mutations mainly affecting the S gene [20]. In some cases, sequencing analysis showed important deletions of the viral genome ranging from small deletions, such as the case of amino-acids (aa) 69–70 of the S protein [21], up to complete or partial deletions of specific viral genes, as observed in some SARS-CoV-2-infected patients in Singapore who displayed, for the first time, complete deletion of the ORF8 gene [22,23]. In SARS-CoV-2, ORF8 is involved in multiple processes [24]. ORF8 is a 121 aa protein with an N-terminal signal sequence mostly unstructured followed by an Ig-like fold [25], which is expressed at the level of the endoplasmic reticulum where it causes ER stress [26]. The ORF8 protein is also secreted as a dimeric form and elicits a pro-inflammatory role stimulating the IL17 receptor [27]. Quite recently, ORF8’s role in the disruption of epigenetic regulation via histone mimicry was demonstrated [28]. Overall, ORF8 appears to be widely involved in immune evasion by downregulating the expression of major histocompatibility complex class 1 (MHC-I) [29] and by suppressing type 1 interferon antiviral response [26]. In addition, ORF8 dysregulates the TGF-β pathway, leading to complications of severe pulmonary diseases, such as lung fibrosis and edema [30].

On the other hand, SARS-CoV-2 open-reading frame 7b (ORF7b) encodes for a protein of 43 aa sharing more than 80% similarity with SARS-CoV-1 [31], and it plays a role in the downregulation of interferon production. Interestingly, a study reporting a deletion of 382 aa (Δ382) located between ORF7b and ORF8 shows that this mutation produces a truncated form of ORF7b with a consequent loss of function of encoded protein [32]. The ORF7b protein structure has not been fully elucidated but is supposed to be a single-pass transmembrane protein acting as a viroporin in a multimeric structure [33]. Small and big deletions in SARS-CoV-2, as seen during previous similar epidemic events, are common and principally found in hot spot regions like the region involved in our deletion [34,35].

The detection of extensive deletions in SARS-CoV-2 specific genes indicates that their loss is not detrimental to the virus’s replication cycle. ORF8 is a gene correlated with escape from the immune system [29], and its absence does not compromise the viral biology, although this genetic loss might lead to a decrease in viral fitness [22]. In this study, we report the detection of six cases with the same deletion of ORF7b and ORF8 in SARS-CoV-2-positive patients screened in northern Italy.

## 2. Materials and Methods

### 2.1. Sample Collection, RNA Extraction, and Quantification

A total of 213 nasopharyngeal swab samples (Copan, Brescia, Italy) were collected from health care personnel, hospitalized patients, and patients entering the emergency room of the AOUI Hospital in Verona, Italy, between August and October 2022. RNA extraction was performed with a Nimbus apparatus (Seegene, Seoul, Republic of Korea) following the manufacturer’s instructions. This study is a retrospective study on anonymized samples submitted to routine diagnosis analysis. It was conducted on anonymized samples, according to the rules established by the ethics committees for clinical trials of the provinces of Verona and Rovigo on retrospective studies. In particular, we analyzed anonymous genetic sequences obtained from samples for routine diagnostic purpose in a retrospective manner. According to Italian regulation, no specific approval is requested. 

### 2.2. Next-Generation Sequencing and Analysis

Quantitative reverse transcription–polymerase chain reaction (RT-PCR) was performed with a Bio-Rad CFX 96 System (Bio-Rad Laboratories, Inc., Hercules, CA, USA), using a commercial kit represented by Allplex SARS-CoV-2 Assay (Seegene, Seoul, Republic of Korea). We proceeded with NGS sequencing only for samples having a cycle threshold value under 32 to maximize sequencing quality.

Library preparation was performed with Illumina COVIDSeq Assay (Illumina, San Diego, CA, USA) with the ARTIC v4 primer pool. Samples were sequenced with the Illumina MiSeq instrument in paired-end mode (2 × 151 bp) with V3 chemistry. The sequence analysis was conducted running a custom pipeline using SAMtools, version 1.18 [36], and Minimap2, version 2.17 [37], on the Linux command line with a minimum depth of 30, minimum mapping quality of value 30 and maximum call fraction at 0.9 as standard parameters for all the sequences. Specifically, the pipeline’s workflow consists in aligning the two pair-ended sequences for each sample via Minimap2; then, it performs the clipping of the primers and the sorting of the aligned reads using SAMtools. After that, the consensus is generated as well as the BAM and BAM.BAI files. Pangolin COVID-19 Lineage Assigner [38] and the Nextclade tool by Nextstrain [39] were used to identify mutations and lineages. Further control of sample read distributions was manually performed using the Integrative Genomics Viewer (IGV) tool [40]. For the alignment of multiple reads with the SARS-CoV-2 reference genome (NC_045512.2), Clustal Omega [41] (EMBL-EBI) version 1.2.4 was employed.

### 2.3. Sequence Comparison and Phylogenetic Analysis

ORF8-deleted viral strain identification was performed by NCBI BLASTn, studying the full-length SARS-CoV-2 sequences deposited in the NCBI and GISAID databases. The design of the phylogenetic tree was created with UCSC UShER [42] and the ETE 3 Toolkit [43].

### 2.4. RNA Secondary Structure Prediction

RNA secondary structures were predicted using the RNA Folding Form version 2.3 from the Mfold web server [44]. The RNA sequence was analyzed as linear with folding temperature fixed at 37 °C and with 1 M NaCl and no divalent ions as ionic conditions. The percent suboptimality number was set to 5, which is the standard offset. The maximum interior/bulge loop size and its maximum asymmetry were also left to default offset, being 30 for both parameters. No limits were given while calculating the maximum distance between paired bases.

The structures were predicted for the ORF7b and ORF8 genes region of Omicron BA.5.1 flanked by 50 nt upstream and 50 nt downstream sequences for a total length of 604 nt.

### 2.5. RNA Retro-Transcription, Amplification and Gel Electrophoresis

RNA extracted from our patients, carrying the ORF7b/ORF8 deletion of 426 nt, was retrotranscribed using iScript Reverse Transcription Supermix for RT-qPCR (Bio-Rad Laboratories, Inc.,, Hercules, CA, USA). The protocol used was based on the manufacturer’s instruction but with some adjustment due to the poor amount of viral RNA in our samples. More in detail, 2 mL of iScript RT Supermix 5X with RNase H+ was mixed with 8 mL of viral RNA extracted, and this reaction mix was incubated in a thermal cycler for 5 min at 25 °C for priming, 20 min at 46 °C for reverse transcription and 1 min at 95 °C for RT inactivation.

The obtained cDNA was amplified with primers flanking the 426 nt deletion, as previously described [15], and with primers inside the deleted region designed with Primer3web version version 4.1.0 [45] online software: ORF8int-F (5′-ATGAAATTTCTTGTTTTCTTAGGAATCATCA-3′) and ORF8int-R (5′-GATGAAATCTAAAACAACACGAACG-3′). Amplifications were performed with GoTaq^®^ G2 Flexi DNA Polymerase (Promega, Madison, WI, USA) following manufacturer’s protocol. Briefly, the reaction of amplification was prepared containing 27.05 mL of nuclease-free water, 10 mL of 5X Colorless GoTaq^®^ Flexi Buffer, 2.4 mL of MgCl_2_ Solution 25 mM, 1.5 mL of PCR Nucleotide Mix 10 mM each (Promega), 2.4 mL of Forward primer solution 10 mM, 2.4 mL of Reverse primer solution 10 mM, 0.25 mL of GoTaq^®^ G2 Flexi DNA Polymerase (5 u/μL), and 4 mL of template cDNA. Reaction mixtures were placed in a thermal cycler with the following amplification protocol: 2 min at 94 °C for Taq activation, which was followed by 35 cycles of 30 s at 95 °C, 40 s at 54 °C, 30 s for 72 °C and a final extension at 72 °C for 5 min. 

The expected size of bands from PCR conducted with primers flanking the 426 nt deletion were 1140 nt or 714 nt for wt or the deleted variant, respectively. For PCR conducted with internal primers, the size of the expected band was 363 nt, while its absence indicates no amplifications and the presence of the 426 nt deletion.

Then, 5 mL of each PCR product was separated on a 2% agarose gel in TAE 1X running buffer and pre-stained with GelRed^®^ Nucleic Acid Staining (Biotium, Fremont, CA, USA). Bands were visualized on a UV Transilluminator (UVidoc HD6 by UVITEC, Cambridge) and analyzed by UVITEC-1D Software version 17 (UVITEC, Cambridge, UK).

### 2.6. PCR Clean-Up and Sanger Sequencing

To perform Sanger sequencing of the PCR product obtained with flanking primers, we proceeded to clean up the remaining 45 mL of PCR product for each sample. Clean-up was performed with NucleoSpin Gel and the PCR Clean-up kit (Macherey-Nagel, Düren, Germany) following the manufacturer’s protocol. Briefly, 45 mL of PCR product was mixed with 90 mL of a 30% NTI buffer solution to remove primer dimers and loaded into a NucleoSpin^®^ Gel and PCR Clean-up column, centrifuged at 11,000× *g* for 30 s, and flow-through was discarded. The column was washed twice with 700 mL of NT3 washing solution with centrifugation at 11,000× *g* for 30 s; in order to better remove the NT3 buffer from the silica membrane, we centrifuged at 11,000× *g* for 1 min and placed the column into a new tube. The silica membrane was dried at 70 °C for 5 min, and DNA was eluted in 30 mL of nuclease-free water preheated at 70 °C by centrifugation at 11,000× *g* after 1 min of incubation. For each sample, we quantified 1.4 mL of recovered DNA with Nanodrop 2000 (Thermo Fisher Scientific Inc., Waltham, MA, USA). Subsequently, 5 mL of a 25 ng/mL DNA sample dilution was mixed with 5 mL of 5 mM primer dilution and delivered to GATC–Eurofins for Sanger sequencing. Sequencing for each sample was performed with external forward and reverse primers. Chromatograms were visualized and analyzed by SNAP Gene Viewer by Dotmatics and aligned with the reference sequence with Codon Code Aligner software version 11.0 by Codon Code Corporation (Centerville, MA, USA).

## 3. Results

### 3.1. Determination of Six Cases of SARS-CoV-2 with 426 nt Deletions in the ORF7b and ORF8 Regions

We identified six SARS-CoV-2-infected patients carrying a 426 nt in frame deletion in ORF7b and ORF8 from routine next-generation sequencing analysis between August and October 2022 at AOUI Verona. 

**Figure 1:**
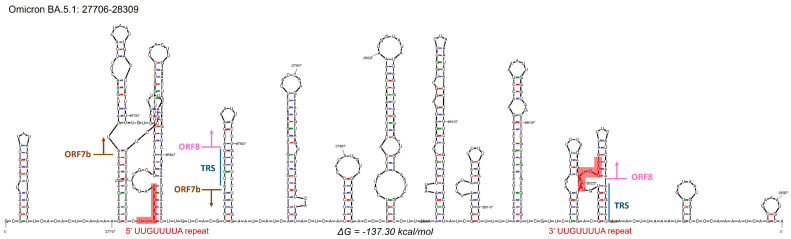
RNA secondary structure with predicted lowest Gibbs free energy (ΔG = −137.30 kcal/mol) of Omicron BA.5.1 variant sequence on the region involved in the deletion. Here are highlighted in red the breakpoints of our deletion, in blue the TRSs, arrows indicate the start and end of ORF7b and ORF8.

All six cases displayed the Omicron BA.5.1 lineage (Pango Lineage, clade 22B defined by Pangolin COVID-19 Lineage Assigner and the Nextclade tool of Nextstrain) with evidence of a consecutive stretch of nucleotide detection failure in the sequence spanning from the ORF7b and ORF8 genes. To confirm that the ‘N’ stretch inserted by Nextstrain was due to a large deletion and not to a drop in the reads coverage, we checked the reads distribution in our sequences using the IGV tool. We observed a deletion of 426 nt at position 27821 (deletion 27821–28247) based on the SARS-CoV-2 NCBI reference genome (NC_045512.2). This rearrangement is in frame and involves the second half of ORF7b and almost the whole sequence of ORF8, presumably generating a fusion protein between the first 22 aa of ORF7b and the last three aa of ORF8. The deletion also includes a putative transcription regulatory sequence (TRS) located between the two ORFs. Pereira [46] deeply analyzes the ORF8 sequence and its flanking sequences in order to understand the mechanism supporting deletions due to rearrangements in the presence of repeats, highlighting the presence of flanking repeats in all deletions of the ORF8 sequences analyzed. It is well known that short repeats could lead to rearrangements and deletions due to discontinuous reading by the RdRp during subgenomic RNA transcription [47]. Interestingly, in our sequences analyzed, the sequence 5′-TTGTTTTA-3′ is present at the two termini of the deleted region. To confirm our hypothesis on the mechanism, we have simulated the RNA secondary structure of the Omicron BA.5.1 variant with the online software RNA Folding Form version 2.3 from the Mfold web server (Figure 1). In order to consider the influence of flanking sequences, we simulated the secondary structure for a region that includes 50 nucleotides flanking regions upstream and downstream the ORF7b/ORF8 deleted region. The RNA-predicted structure of these regions, simulated at 37 °C, highlights multiple hairpins, which could facilitate genetic rearrangements like strand transfer [48]. The variation in the Gibbs free energy value (ΔG = −137.30 kcal/mol), calculated for the formation of this secondary structure, indicates that these hairpins can take place with high probability. The RNA sequence was analyzed as linear with folding temperature fixed at 37 °C and with 1 M NaCl and no divalent ions as ionic conditions. The percent suboptimal number was set to 5, which is the standard offset. The maximum interior/bulge loop size and its maximum asymmetry were also left to default offset, being 30 for both parameters. No limits were given while calculating the maximum distance between paired bases.

### 3.2. Sanger Analysis Confirms the Deletion and Its Consistency

**Figure 2:**
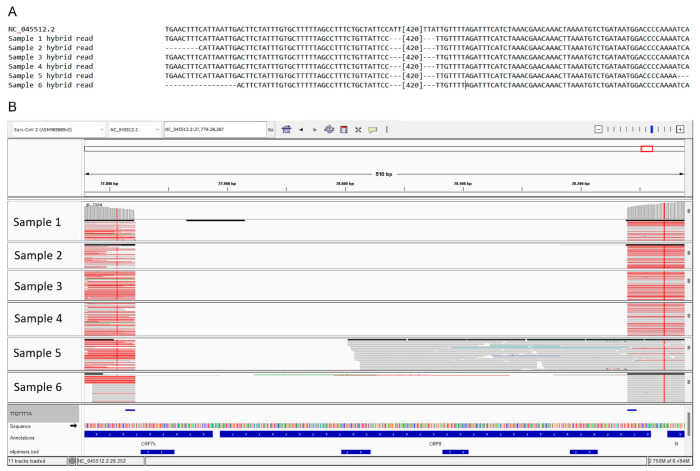
Alignments of reads generated by NGS sequencing. (**A**) Alignment of patients’ reads with the reference sequence; (**B**) IGV visualization of the single reads for each sample. Red lines indicate hybrid reads.

We paired the reads covering the region using the Clustal Omega alignment tool and observed the presence of hybrid reads formed by a combination of nucleotides aligned upstream and downstream of the deleted region (Figure 2, panel A and B).

**Figure 3:**
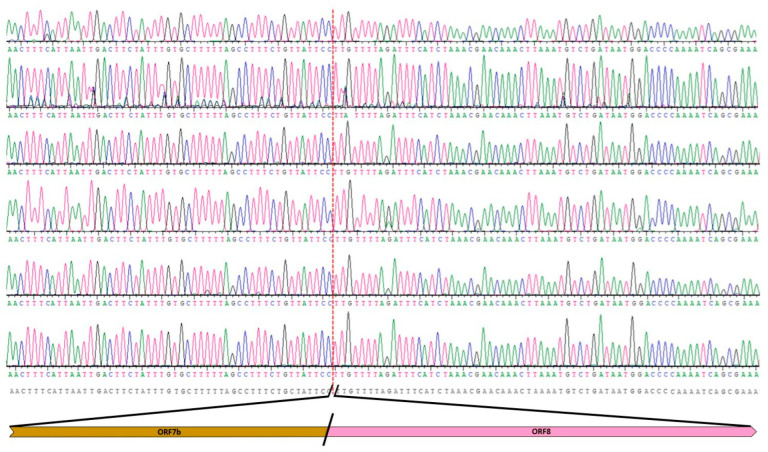
Chromatograms alignment of the Sanger sequencing product with the reference genome. Chromatograms were obtained from PCR reactions from our six samples. Red lines show the breakpoint site and in the lower part of the figure it is shown the deleted nucleotides with the indication of the ORF.

We confirmed this observation using the classical Sanger procedure through the design of two oligonucleotides upstream and downstream of the deletion region to amplify a product of 832 bp for the wild-type sequence or 406 bp for the deleted ones. These amplicons were sequenced using the Sanger procedure, and the nucleotide analysis demonstrated the absence of 426 nt in all six samples (Figure 3). 

### 3.3. Phylogenetic Analysis of SARS-CoV-2 Strains with 426 nt Deletions

Starting with the detected hybrid reads as a query, we investigated the presence of similar sequences in the NCBI database (BLASTn tool), and the results showed 15 samples with comparably sized deletions of the ORF7b/ORF8 region, in some cases, with slightly different breakpoints. In seven of 15 samples, a best alignment score with our queries was detected, carrying the same deletion of 426 nt. The Omicron variant (clade 22B) was identified in seven of 15 cases and classified as BA.5.1; six were classified as clade 20A, variant B.1.243; and two were classified as the Delta variants AY.103 and AY.58.

**Figure 4:**
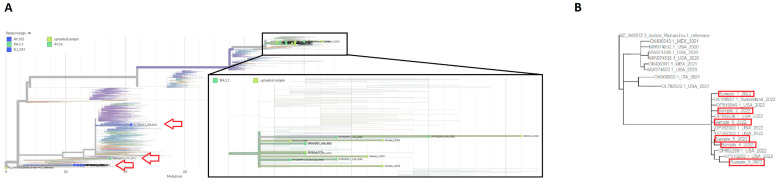
Phylogenetic tree. (**A**) UShER phylogenetic analysis of the sequences with similar size deletions of ORF7b and ORF8. Arrows indicate the locations of sequences carrying a big deletion of ORF8. In the enlargement, branches with our 6 patient samples are shown; (**B**) Phylogenetic analysis specific for the 15 strains having ORF8 deletion found on NCBI and our sequences. Red boxes indicated our six sequences.

To evaluate the evolutionary distances between the sequences carrying large deletions of ORF8, we performed phylogenetic analysis (Figure 4A,B). The phylogenetic tree designed by UShER underscored that those similar deletions appeared multiple times during the evolution of the virus (indicated by arrows), but all the Δ426 mutants appeared in 2022. The simultaneous appearance of the same deletion in the last few months appears unlikely. In the enlargement, we show the branch with our samples. 

### 3.4. Clinical Context of Patients Carrying the Δ426 Mutation

General information (age, sex, etc.) of patients carrying the Δ426 mutation is shown in Table 1. All patients were infected between August and October 2022, and three of six patients were immunosuppressed or immunocompromised according to criteria previously published [49], and four of six patients exhibited at least one comorbidity.

## 4. Discussion

While single-nucleotide polymorphisms (SNPs) and small deletions were extensively studied and monitored [50,51,52], relatively little is known about large deletions (>100 nt), which can easily be misinterpreted as a lack of coverage by automated tools for sequencing data analysis [53]. In this study, we describe a consistent deletion of 426 nt detected in six samples from nasopharyngeal swabs of SARS-CoV-2 infected patients, causing a truncated ORF7b and loss of ORF8 transcripts, removing the transcriptional regulatory sequence located between the ORF7b and ORF8 genes.

It is well known that ORF7b and ORF8 are located in a genome region characterized by a high mutation rate and thus considered a mutational hotspot. Genetic mutations in this region might correlate with zoonotic events and pandemic waves [54,55]. SARS-CoV-2 ORF7b encodes for a 43 aa protein showing more than 80% similarity with its SARS-CoV-1 homolog [31], and it plays an important role in interferon downregulation [56]. Its structure has not been fully elucidated, but it has been suggested that this protein could be considered a single-pass transmembrane protein acting as viroporin with a multimeric structure [33,57]. 

The SARS-CoV-2 ORF8 gene is common to several SARS-related coronaviruses [46], but it is highly divergent from its homolog in SARS-CoV-1 [58]. The ORF8 gene encodes for a 121 aa accessory protein with an N-terminal transmembrane peptide followed by an Ig-like domain [25]. The ORF8 protein is expressed at the endoplasmic reticulum (ER) level, where it causes ER stress [26], and its dimeric form elicits a pro-inflammatory role, stimulating the IL-17 receptor [27,28]. A recent study [59] demonstrated that ORF8 can induce ER reshaping by its involvement in the formation of mixed disulfide complexes with ER proteins. This remodeling is advantageous for ORF8 to escape from degradation, and it activates ER stress, alters ER homeostasis, and accelerates protein trafficking through the ER. Moreover, ORF8 is also involved in the disruption of epigenetic regulation via histone mimicry and the degradation of MHC-I [29]. Overall, ORF8 appears to be widely involved in immune evasion by suppressing the type 1 interferon antiviral response [26] and deregulating the TGF-β pathway [46]. It was speculated that the acquisition of a new ORF8 via recombination could play a consistent role in transmission from animals to humans [60]. 

A discrete number of genomic deletions in ORF8, ranging from 1 to 382 nt [58,60,61], as well as truncated forms [23,61], have been reported for SARS-CoV-2 (see graphical representation in Supplementary Figure S1). The partial or complete loss of ORF8 was also reported for SARS-CoV-1, with some sequences exhibiting a 415 nt deletion similar to our 426 deletion [62], and such losses could be involved in the zoonotic transition, favoring an adaptive change [62]. Half of the mutations within the ORF8 gene, identified in the first 6 months of the pandemic, were detrimental for the encoded protein [63]. More frequent mutations include nonsense mutations (for instance, Q27*) [23] and deletion events, some of which completely abolished the expression of the gene product [22,53]; in addition, extensive deletion might involve upstream ORF7b and, in some cases, even ORF7a [64]. In SARS-CoV-2, ORF8 is involved in many processes and seems to be far from dispensable in vivo [24]. Massive deletion events do not represent a novelty for the Coronaviridae because this phenomenon was already described for the homolog in the variant of SARS-CoV-1 at the end of the epidemic era [62]. Interestingly, a study of a deletion of 382 nt (Δ382) showed that this mutation produces a truncated form of ORF7b, abolishing its activity [62]. 

According to the literature [46], hairpins and repeats in the ORF7b/ORF8 transcript region, as predicted for BA.5.1 variant sequence, could play a role in genomic rearrangements during viral genome replication [59]. An abundant formation of hairpin structures in the ORF7b and ORF8 sequence could facilitate the rearrangement events driven by short repeats. The so-called Δ382 variant, first described in Singapore in January/February 2020, was reported to induce milder clinical symptoms compared to the wild-type SARS-CoV-2 strains [22,23]. 

In this paper, we describe a consistent deletion found in six samples from infected patients that truncates ORF7b, abolishes the ORF8 transcripts, and removes the TRS located between the ORF7b and ORF8 sequences, as shown in Figure 1. The analysis with Swiss-Model [65] and Protter [66] of a hybrid protein generated by 426del suggests a single alpha-helix secondary structure and transmembrane localization. The truncated peptide seems to retain the transmembrane domain, although in a curiously inverted configuration (Supplementary Figure S2), with the N-terminal inside the membrane. Due to a lack of knowledge about the ORF7b protein and the small dimension of our hybrid, its role in SARS-CoV-2 infected patients carrying the 426 deletion remains unknown. Liu and coworkers [59] demonstrated that the use of reducing agents supported the release of ORF8 from mixed disulfide complexes, favoring its degradation and resolution of ER stress. 

Our six cases showed particular clinical and viral genome characteristics: all cases shared the BA.5.1 variant, confirming some similar observations from Germany and Switzerland; and three of six patients displayed the presence of immunodeficiency or autoimmune disease, whereas the remaining three cases did not show evident immunological disorders. Immune system derangement generally allows for a larger number of in vivo viral replication cycles, thus determining a longer time of infection with the onset of more frequent genome mutations or deletions. Strikingly, our six cases exhibited a persistence of infection even in immunocompetent patients. The absence of ORF7b and ORF8 might enable the deleted mutation to escape from the interferon response and innate immunity; however, we have no information about the replication and transmission rate effectiveness of our mutations. 

Although variants with ORF7b and ORF8 region deletions have been described to different extents during the infection waves, the number of sequenced ORF7b/8 deleted strains is very small, and in some cases, these deletions were present in the same patient, with the full-length variant suggesting the evolution of a viral strain [27]. In addition, we should take into account that these large deletions are considered to be rare not because they do not appear frequently, but several sequencing pipelines are not able to automatically detect these deletions, thus determining an underestimation of phenomenon. Starting from this observation and according to the studies by Zinzula and DeRonde and coworkers [61,67], we speculate that this deletion might be considered a possible pathway for viral evolution, which appeared during the succession of the different variants as an alternative route for viral spreading, although its development and evolutionary success might be overcome by the continuous and rapid appearance of new variants with better transmissibility and replication. Moreover, it should be noted that the low frequency of this deletion could be related to other cases of functional abolishment of ORF8 (as the already mentioned stop codon Q27* and the more recently appeared Q18* and G8*). Hisner and colleagues [68] have also noticed that the BA.5 variants are characterized by a mutation in the TRS (C27889T) that probably interferes with a proper generation of the ORF8 subgenomic RNA and thus with its expression. Interestingly, Hisner [68] hypothesized an evolutionary trend which moves toward a functional disappearance of ORF8 expression starting with BA.5 variants and carrying on with the XBB variants bearing the G8* mutation. Hence, it is highly probable that the 426 nt deletion was not able to overcome the competition of other variants that were de facto deleted for these gene. Notwithstanding the small impact of these mutations, the onset of ORF7b/8 deleted, or functionally deleted, mutant variants is playing a role in the evolutionary balance between host and virus.

## 5. Conclusions

The genomic surveillance of SARS-CoV-2 variants with the next-generation sequencing technique, carried out on a global scale and shared via the GISAID database, has determined the complete analysis of genetic changes of SARS-CoV-2. The sequencing of the total viral genome has highlighted the presence of numerous point mutations, substitutions, deletions and insertions, as well as more extensive ones, leading to the complete elimination of entire gene portions. At an evolutionary level, it is known that RNA viruses mutate consistently to find a sort of biological balance with its host.

The loss of ORF8 is detected in the current circulating variants XBB.1.5, XBB.1.9 and XBB.1.16 and their sublineages. The gene portion of ORF8 has a stop codon in the eighth aa, leading in itself to the lack of the encoded protein. These sublineages displayed a lower clinical impact, guaranteeing the fitness of the virus. 

The patients carrying the 426 nt deletion showed a milder phenotype compared to the symptoms of COVID-19 pneumonia. This could be traced back to the fact that the ORF8 protein contains an Ig-like domain and is highly immunogenic; therefore, its absence might be linked to less severe COVID-19, lower risk of hypoxia and less systemic infection.

Further experiments are necessary to verify which cellular pathways are altered due to the deletion described here. The data obtained will help to better understand the function of ORF7b and ORF8 and to choose the most suitable pharmacological strategy.

## Figures and Tables

**Table 1:** Patient data.

ID	Age	Sex	Sequencing Date	Length of Viral Genome	Relevant Pathology	COVID-19 Treatments	Other Treatments
1	59	F	October 2022	29,353 nt	RA, PV, RSV coinfection, bacterial pneumonia	Tixagevimab + Cilgavimab	Methylprednisolone
2	64	F	October 2022	29,320 nt	AKI, peritoneal carcinomatosis	None	Carboplatinum
3	52	F	October 2022	29,331 nt	None	None	None
4	74	M	October 2022	29,321 nt	Non-Hodgkin lymphoma	Tixagevimab + Cilgavimab	R-COMP
5	83	F	October 2022	29,086 nt	Aspiration-associated pneumonia	Remdesivir	None
6	54	F	August 2022	29,328 nt	None	None	None

## Data Availability

Data supporting reported results can be found in GISAID database (https://gisaid.org/ accessed on 22 September 2023) identified as: EPI_ISL_15367204 (Sample 1), EPI_ISL_17796641 (Sample 2), EPI_ISL_15505746 (Sample 3), EPI_ISL_16196755 (Sample 4), EPI_ISL_16233780 (Sample 5) and EPI_ISL_14493878 (Sample 6).

## Supplementary Materials

The following supporting information can be downloaded at: https://www.mdpi.com/article/10.3390/microorganisms11102378/s1, Figure S1: Schematic representation of SARS-CoV-2 (color filled) and SARS-CoV-1 (striped) genomic sequences carrying ORF7a, ORF7b and/or ORF8 deletions. In the enlargement are shown sequences carrying big deletions compared to Wuhan strain and our 426del. From top to bottom, are represented: the reference sequence NC_045512.2; 426 nt deletion found in this study; 382 nt deletion found in Singapore in 2020; 138 nt deletion found in Australian samples in 2020; 345 nt deletion found in Bangladesh samples in 2020; 62 nt deletion found in Spanish samples in 2020; 872 nt deletion found in Poland in 2021; 415 nt deletion found in SARS-CoV-1 in 2023. In yellow are shown the TRS. In sky-blue is represented ORF6 gene, in blue, ORF7a gene, in brown ORF7b gene, in pink is shown ORF8 gene and in green N gene. Deletions are represented as line: the 426 deletion here described is in red while others SARS-CoV-2 deletions are in blue. The last genome here represented is SARS-CoV-1 genome with a 415 nt deletion spanning between ORF7b and ORF8. Figure S2: Protter predictions. (A) Prediction of ORF7b protein; (B) the hybrid peptide product resulting from the ORF7b and ORF8 426 deletion. Interestingly, the conformation of the peptide has predicted to be in an inverse configuration, with the N-terminal inside the membrane.
