# Supplementary figures and images for: Detection of SARS-CoV-2 Δ426 ORF8 Deletion Mutant Cluster in NGS Screening
Source: Microorganisms. 2023 Sep 23;11(10):2378. doi: 10.3390/microorganisms11102378 (PMC10609088; doi:10.3390/microorganisms11102378)

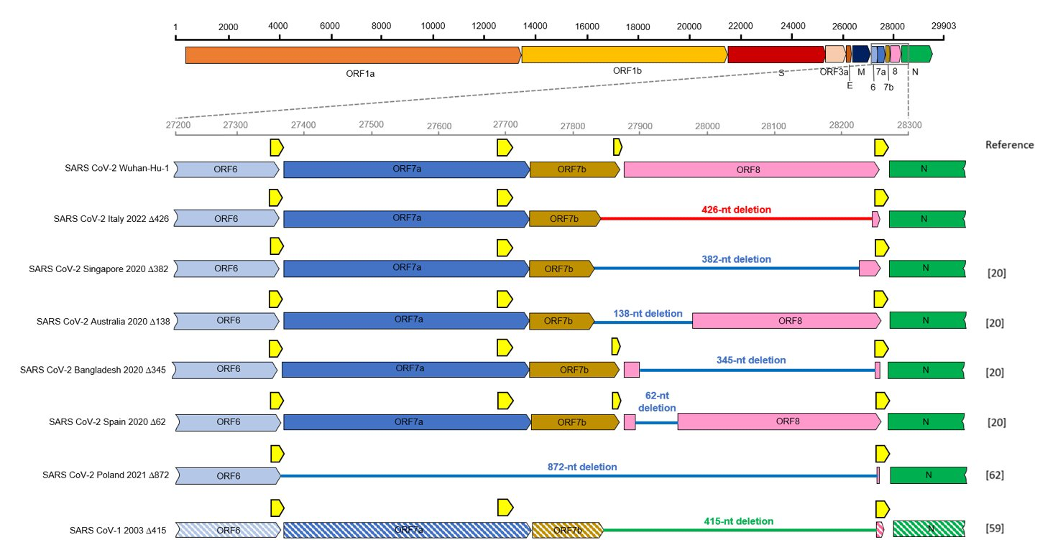

Supplement: Supplementary file 1 [file microorganisms-11-02378-s001.zip › Suppl Fig 1.png]

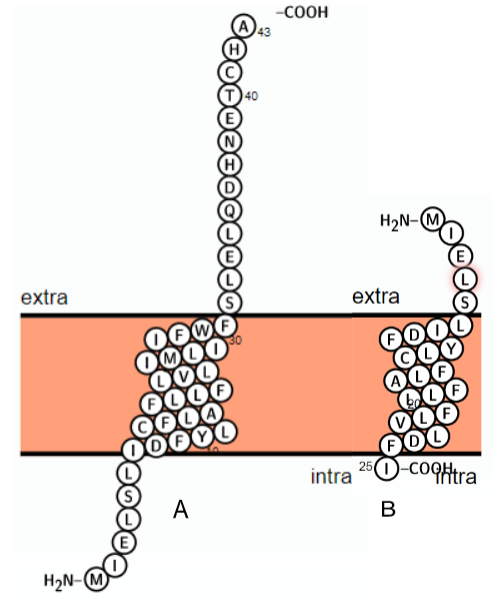

Supplement: Supplementary file 1 [file microorganisms-11-02378-s001.zip › Suppl Fig 2.png]
